# Inducible immortalized Dendritic Cells enable antigen-specific antibody production in a murine *in vitro* Immunization model

**DOI:** 10.1371/journal.pone.0339883

**Published:** 2026-01-02

**Authors:** Juliane Egert, Natalia Maier, Burkhard Micheel, Katja Hanack

**Affiliations:** Chair of Immunotechnology, Institute of Biochemistry and Biology, University of Potsdam, Potsdam, Germany; University of Arizona, College of Medicine-Phoenix, UNITED STATES OF AMERICA

## Abstract

Transferring the complexity of the *in vivo* immune response into a controlled *in vitro* system represents a promising strategy for the rapid generation of antigen-specific antibodies while significantly reducing the reliance on animal experimentation, thus adhering to the 3Rs principle. In this study, we present an *in vitro* immunization (IVI) approach that employs conditionally immortalized dendritic cells (DCs), derived from a transgenic irtTA-GBD/T-Ag mouse model, to initiate antigen-specific immune responses *in vitro*. These dendritic cells can be kept in a proliferative state under tetracycline-controlled expression of the SV40 large T antigen (termed iniDCs) and maintained long-term in culture. Upon de-induction of the immortalizing transgene, the resulting deinduced iniDCs (de-iniDCs) regain physiological properties and exhibit full functional maturation in response to appropriate stimuli. When co-cultured with naïve murine T and B lymphocytes, these mature de-iniDCs are capable of initiating antigen-specific adaptive immune responses, culminating in the production of antigen-specific antibodies by B cells. By utilizing this standardized population of antigen-presenting cells, we have established a robust and reproducible IVI protocol that enables the generation of antigen-specific antibody responses *in vitro*. Antibody-secreting B cells were subsequently fused with myeloma cells, and antigen-specific hybridomas were rapidly identified and isolated using the novel *selma* (*selection of monoclonal antibody*) technology, allowing for an accelerated and efficient selection of antigen-specific monoclonal antibodies.

## Introduction

The induction of antigen-specific immune responses *in vivo* involves a well-regulated interaction of several immune cell types, such as dendritic cells (DCs), T and B cells. Putting the involved cell types in an *in vitro* setup allows a mimicry of the *in vivo* conditions to carry out an *in vitro* immunization (IVI). Such an IVI is of special interest since the demand for particular binding molecules such as antibodies increases rapidly for research, diagnostics, and therapy.

The first approaches have been successfully carried out relying on the interaction of DCs as antigen-presenting cells (APCs) with naïve T cells that polarize into Th2 effector cells and activate B lymphocytes to proliferate and produce antigen-specific antibodies [1–3]. Those B cells were then fused with myeloma cells to obtain hybridomas, followed by a conventional screening process according to the state-of-the-art hybridoma technology published by Köhler and Milstein [4]. Although this process is an improvement in time and needs less antigen, it does not finally abolish the use of laboratory animals. Since immune cells are short-living primary cells, they only survive for a few days in cell culture. In addition, not all required cell populations can be isolated from the same animal. Bone marrow-derived DCs (BM-DCs) are generated *in vitro* over 7 days out of hematopoietic stem cells [5,6]. Therefore, the isolation of T and B lymphocytes requires a second animal of the same genetic background, and the actual protocol of an IVI requires at least two mice. However, the isolated cell populations are highly heterogeneous, and cell viability and condition may differ between the single animals.

To circumvent the problem of short-living heterogeneous immune cell populations and to reduce the application of laboratory animals, it would be an essential improvement to generate immortalized cells that can be used as a scaffold for standardized reproducible experimental conditions, but the immortalization of fully differentiated primary cells remains challenging. The insertion of tumor suppressor genes [7–9] led to a stable immortalized splenic DC-line DC2.4 [10,11] that is still functional but not freely available. The integration of oncogenes [12,13] also led to immortalized DCs [14], but their expression must be tightly controlled, and the consequences for possible cellular malfunctions remain unknown. Growth-factor-dependent long-term cultures [15,16] have only been developed from splenic DCs. To our knowledge, no functional immortalized DC cell line is available that enables an *in vitro* immunization.

In this study, we used the double transgenic irtTA-GBD/T-Ag mouse strain that incorporates the large T-antigen (T-Ag) under the control of an improved TetOn® system to generate a long-living DC population that can be reversibly immortalized [17,18]. Following the protocol from Richter et al. [19], cells were transferred into an immortal period by simultaneous addition of the glucocorticoid dexamethasone (Dex) and the tetracycline doxycycline (Dox) and expanded *in vitro*. During this immortal period, they lost their specific dendritic phenotype but started to proliferate again, as shown by a CFSE staining. Upon deinduction, the generated de-iniDCs restored their morphology and were capable of engulfing, processing, and presenting our recently introduced model antigen VP1 [20,21] similarly to primary bone marrow-derived DCs (BM-DCs).

**Fig 1:**
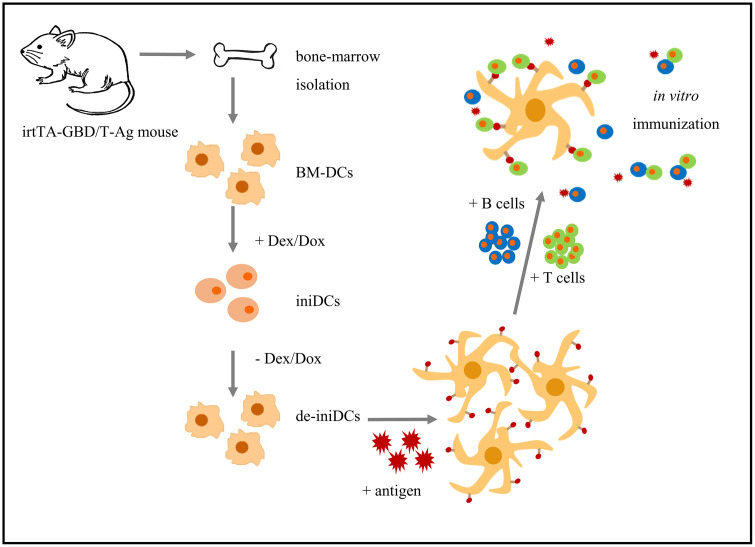
Schematic overview of the isolation process of immortal dendritic cells (DCs), their maturation, and the following *in vitro* immunization. BM-DCs = bone marrow derived DCs, Dex = dexamethasone, Dox = doxycycline, iniDCs = induced-immortal DCs, de-iniDCs = deinduced iniDCs.

When using this immortal DC population in our IVI protocol (Fig 1), we could establish a reproducible IVI approach which also allows a considerable reduction of laboratory animals.

## Methods

### Animals

For experiments, 8–12 week-old mice were used. C57Bl/6 and transgenic irtTA-GBD/T-Ag mice were bred in our facility at Potsdam University (Potsdam, Germany). Double transgenic irtTA-GBD/T-Ag mice with a C57Bl/6 background (Immorto mouse) were a kind gift of K. Anastassiadis (Dresden, Germany). Those mice express the SV40 large T-antigen (T-Ag) under the control of a TetOn® system and a codon-optimized reverse tetracycline transactivator (irtTA) fused to the ligand-binding domain of a mutated glucocorticoid receptor under the control of a ubiquitously expressed CAG promotor [17].

### Preparation of murine dendritic cells from hematopoietic stem cells

Bone marrow cells were isolated by flushing the tibia and femur of mice as described [22]. Cells were homogenized with a 40 µm easy strainer (Greiner, Austria), washed in RPMI 1640 (Thermo Fisher Scientific, Waltham, Massachusetts, USA), and resuspended in RPMI 1640 complete medium supplemented with 2 mM L-glutamine (Carl Roth, Karlsruhe, Germany) 50 µM β-mercaptoethanol (Carl Roth, Karlsruhe, Germany), 0.1 mM sodium pyruvate (Sigma Aldrich, Missouri, USA) and 10% heat-inactivated fetal calf serum (FCS, Thermo Fisher Scientific, Massachusetts, USA). Cells were centrifuged at 320 x g, 4°C for 10 min and cultivated in RPMI complete medium containing 40 ng/ml recombinant murine *granulocyte macrophage-colony stimulating factor* (rmGM-CSF) at 37°C and 6% CO_2_. After 7 days, suspension cells were passaged into a new culture flask. The culture medium was refreshed after 2–3 days.

### Isolation and enrichment of Naïve lymphocytes

Naïve lymphocytes were isolated from the spleen of a C57Bl/6 mouse. Isolation and enrichment of naïve lymphocytes were performed using the CD4^+^ CD62L^+^ T cell Isolation kit II and the B cell Isolation kit, both from Miltenyi Biotec (Bergisch Gladbach, Germany). The procedure was carried out according to the manufacturer´s instructions using a magnetic cell sorting device provided by the same company.

### Cultivation of immortal dendritic cells

**Fig 2:**
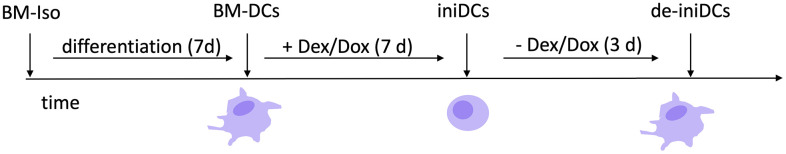
Timeline diagram of cultivation stages of immortal DCs. Hematopoietic cells isolated from the bone marrow (BM-Iso) were differentiated into bone marrow derived DCs (BM-DCs) for 7 days with the addition of GM-CSF. These BM-DCs were transferred into an immortal period (iniDCs) by stimulation with Dex/Dox for at least 7 days. Before further functional studies the stimulus was removed for 3 days (de-iniDCs).

BM-DCs from double transgenic irtTA-GBD/T-Ag mice were isolated as described above. Seven days after isolation, suspension cells were transferred in a new culture dish and stimulated with 100 nM dexamethasone (Dex, Sigma Aldrich, Missouri, USA) and 1 µg/ml doxycycline (Dox, Sigma Aldrich, Missouri, USA) to induce the expression of the T-Ag as well as 40 ng/ml rmGM-CSF to keep up the differentiation status of DCs. Those cells are referred to as induced-immortal DCs (iniDCs). RmGM-CSF was reduced stepwise with every culture medium exchange to 10 ng/ml to reduce the development of granulocytes. Suspension cells were transferred into a new culture dish every 2–3 days. Therefore, suspension cells were harvested by gently flushing the cell culture dish and centrifuged at 320 x g, at 4°C for 10 min. The supernatant was discarded, and cells were resuspended in fresh culture medium added with Dex, Dox and rmGM-CSF as described. Before maturation, iniDCs were cultivated without dex and dox for 3 days to regain their primary cell-like characteristics. After this deinduction the cells are referred to as de-iniDCs (Fig 2).

### Stimulation of DCs with DQ-Ovalbumin

Confirmation of antigen uptake was performed by using self-quenched ovalbumin (DQ-Ova, Thermo Fisher, USA). Only after phagocytosis and proteolytic cleavage, fluorescence is detectable at 510 nm. Respectively, 1 x 10^5^ DCs were stimulated with 1 µg/ml DQ-Ova (Thermo Fisher Scientific, Waltham, Massachusetts, USA), for 60 min at 37°C. Fluorescence was measured by flow cytometry using the Attune® Acoustic Focusing Cytometer (Thermo Fisher, Massachusetts, Germany). For fluorescence microscopy using the BZ-8000 Biozero (Keyence, Neu-Isenburg, Germany), 5 x 10^5^ de-iniDCs were challenged with 2 µg/ml DQ-Ova for 60 min at 37°C.

### Activation of immortal dendritic cells by VP1 antigen

To study the maturation capacity of irtTA-GBD/T-Ag DCs, we used hamster polyomavirus (HaPyV) major capsid protein 1 (VP1) as a model [20,21]. Therefore, 1.5 x 10^6^ de-iniDCs were plated in 2 ml of RPMI complete medium containing 10 ng/ml rmGM-CSF. Cells were stimulated with 15 µg/ml VP1 for 20−22 h at 37°C and 6% CO_2_. The supernatant was collected before and after stimulation to measure the maturation markers Interleukin 12 (IL-12) and IL-10 by using a Mouse IL-12p70 or IL-10 ELISA kit (eBioscience, Germany).

### Flow cytometry analysis of DC maturation

The maturation of immortal DCs was analyzed by flow cytometry using the specific DC activation markers CD11c, major-histocompatibility-complex-II (MHC II), CD40, and CD86 [3]. Therefore, de-iniDCs were challenged with 15 µg/ml VP1 or left untreated. Cells were harvested 20−22 h after stimulation and centrifuged at 320 x g for 10 min at 4°C. The supernatant was discarded, and cells were washed twice in MACS buffer. Respectively, 5 x 10^5^ washed cells were resuspended in staining buffer containing antibodies against either phycoerythrin (PE) conjugated recombinant human anti-mouse-CD11c IgG antibody (Miltenyi Biotec, Bergisch Gladbach, Germany, Ref.: 130-110-701, Lot: 5190521242), rat anti-mouse-CD40 IgG antibody (BD Bioscience, Heidelberg, Germany, Cat.: 55379, Lot.: 8184649), rat anti-mouse-MHC II IgG antibody (Miltenyi Biotec, Bergisch Gladbach, Germany, Ref.: 130-102-186) or fluorescein isothiocyanate (FITC) labeled rat anti-mouse-CD86 IgG antibody (Miltenyi Biotec, Bergisch Gladbach, Germany, Ref.: 130-102-506, Lot.: 5171106113) diluted 1:50 in MACS buffer and incubated for 30 min at 4°C in darkness. Stained cells were again washed twice in MACS buffer and finally resuspended in 300 µl MACS buffer. Before analysis, 1 µg/ml PO-PRO^TM^-1 Iodide (Thermo Fisher, Massachusetts, USA) was added. Cells were measured using the Attune® Acoustic Focusing Cytometer (Thermo Fisher, Massachusetts, Germany). Isotype controls were carried out accordingly. Data from flow cytometry were analyzed using the FlowJo^TM^ Software v10 (BD, Ashland, USA).

### Proliferation analysis of iniDCs

Due to the expression of the T-Ag, DCs isolated from double-positive irtTA-GBD/T-Ag mice can proliferate during their immortal period. Therefore, iniDCs were stained with the cell trace dye carboxyfluorescein succinimidyl ester (CFSE, Thermo Fisher, Massachusetts, Germany) according to the protocol provided by Quah et al. [23]. Briefly, iniDCs were generated as described above and after 7 days of Dex/Dox stimulation, 35 x 10^5^ iniDCs or de-iniDCs were stained with either 5 µM, 7.5 µM, or 10 µM CFSE in PBS or PBS/NKS 5%. Respectively, 5 x 10^5^ iniDCs or de-iniDCs were seeded and analyzed by flow cytometry using the BD FACSDAria III^TM^ (BD Bioscience, Heidelberg, Germany). Analysis of iniDCs was performed at days 0, 1, 6, 8, 11, and 15 and of de-iniDCs at days 0, 1, 2, 3 and 4. Before analysis, iniDCs and de-iniDCs were additionally stained with PE-conjugated recombinant human-anti-mouse-CD11c-IgG antibody (Miltenyi Biotec, Bergisch Gladbach, Germany, Ref.: 130-110-701, Lot: 5190521242) and 1 µg/ml PO-PRO^TM^-1 Iodide (Thermo Fisher, Massachusetts, USA) as described above.

### Cultivation and activation of immune cells for *in vitro* immunization

To detect whether immature de-iniDCs were still able to induce an immune response *in vitro,* 1 x 10^5^ de-iniDCs were seeded in either RPMI complete medium containing 25 ng/ml Flt3-Ligand (Flt3L, Peprotech, Hamburg, Germany) and 10 ng/ml rmGM-CSF or only rmGM-CSF. Cells were activated with 15 µg/ml VP1, 15 µg/ml N-Protein or 15 µg/ml pAS6 as described above. After 20–22 h of maturation, different ratios of naïve CD4^+^ T cells (DC:T, 1:1, 1:2, 1:5, 1:7, 1:10) were added as well as 7 x 10^5^ naïve B cells, both isolated from a C57Bl/6 mouse. In addition to the B cells, immune cells were restimulated with the used antigen. Cells were co-cultivated for 7 days in RPMI complete medium supplemented with 25 ng/ml IL-21 (PeproTech, Hamburg, Germany), 5 µg/ml *Schistosoma egg antigen* (SEA), and 10 ng/ml rmGM-CSF at 37°C and 6% CO_2_. After 7 days of cultivation, the cells from the IVI with VP1 and pAS6 were harvested and fused with murine SP2/0 myeloma cells. Immunized B cells from the IVI with the N protein were fused with transgenic SP2/0 myeloma cells and selected using the novel *selection of monoclonal antibody (selma)* technology developed by our working group [24]. To detect antibody production from the IVI-system over time, culture supernatant was collected at days 3, 4, 5, and 6 and analyzed for IgG-production by ELISA. The removed medium was replaced with fresh RPMI medium, containing SEA and IL-21.

### Electrofusion of *in vitro* activated murine B lymphocytes

On day 7 of the IVI, immunized B lymphocytes were electrofused to SP2/0-Ag14 or transgenic SP2/0-Ag14 [24] myeloma cells in the presence of polyethylene glycol (Carl Roth, Karlsruhe, Germany) as previously described [25]. For the selection process, fused hybridoma cells were seeded in hypoxanthine-aminopterin-thymidine (HAT, VWR, Pennsylvania, USA) containing RPMI complete medium in a 96 flat-bottom well plate onto mouse peritoneal feeder cells for 14 days.

After HAT-selection culture supernatants were analyzed by enzyme-linked immunosorbent assay (ELISA), and positive cultures were seeded in a limited dilution. The resulting clones were regularly checked for antibody production. Antigen-specific antibody-producing monoclonal hybridomas were selected microscopically and expanded stepwise to a T75 cell culture vessel VWR, Pennsylvania, USA) to collect the supernatants up to 500 ml. Antigen-specific polyclones were again separated by limiting dilution and further analyzed regularly for antibody production of monoclonals.

### Detection of antigen-specific antibodies in culture supernatants and the isotype of purified antibodies by ELISA

Screening for antigen-specific antibodies and detection of purified antibody isotypes were performed by ELISA. Therefore, either 5 µg/ml antigen or purified antibody was coated on a 96-well microtiter plate (Greiner, Bio-One GmbH, Germany) in 50 µl PBS overnight at 4°C. After washing with deionized water, free binding spots were blocked for 20 min using Roti®Block (Carl Roth, Karlsruhe, Germany) at room temperature (RT). Plates were washed again, and 50 µl supernatant or biotinylated isotype-detection antibody were incubated for 2 h at RT. After washing, a peroxidase (POD) labeled goat-anti-mouse-IgG(Fc) antibody (Dianova, Hamburg, Germany. Ref.: 115-035-071, Lot.: 149085) or streptavidin-POD (SAV-POD, Roche, Mannheim, Germany) was incubated for 30 min at RT. Plates were washed again and 3,3′,5,5′-tetramethylbenzidine substrate (TMB) was added. The reaction was stopped by H_2_SO_4_ and measured at 450 and 620 nm using the BeckmanCoulter^TM^Detection Platform (Beckman Coulter GmbH, Germany).

### Selection of monoclonal antibodies using *selma* technology

B cells fused with the transgenic SP2/0-Ag14 cell line were screened using the *selma* technology [24]. The transgenic SP2/0-Ag14 cells possess an artificial cell surface receptor that enables a fast and straightforward enrichment of antigen-specific antibody-producing cells using fluorescence-activated cell sorting (FACS) [24].

Briefly, hybridoma cells were collected after HAT selection and incubated at 1:50 with an antibody-capture-matrix (ACM) that consists of an anti-mouse IgG/IgM antibody coupled to streptavidin (SAV). The ACM binds to the biotinylated artificial cell surface receptor of the transgenic SP2/0 cells and captures isotype-specific the released antibodies from the hybridoma cells. Next, the complex was incubated with 1 µg antigen per 1 x 10^6^ cells, to select antigen-specific antibody-producing hybridomas. Finally, a fluorophore-conjugated goat anti-Histidine tag detection antibody (Bio-Rad, Munich, Germany, AHP1656F) was added at twice the concentration of the antigen, and single cells were analyzed using the BD FACSDAria III^TM^ (BD Bioscience, Heidelberg, Germany). Antigen-specific antibody-producing hybridomas were sorted onto a 96-well microtiter plate in a single-cell deposition and tested for antibody production by ELISA.

### Purification of murine monoclonal IgG antibodies

Culture supernatants from murine hybridoma cells were purified as previously described [25,26]. Briefly, supernatants were filtered, mixed with binding buffer (4 M NaCl, 1 M glycine NaOH, pH 8.5), and loaded on a protein A column. Bound IgG antibodies were eluted with 0.1 M citrate (pH 5.0) and neutralized with Tris-HCl (pH 9.0), then concentrated with an Amicon^®^ Ultra Centrifugal Filter (MWCO 30kDa, Merck, Darmstadt, Germany) and analyzed by SDS-PAGE and ELISA.

### Sodium dodecyl sulfate-polyacrylamide gel electrophoresis (SDS-PAGE)

SDS-PAGE was applied to verify the purification of the monoclonal antibodies from the hybridoma cell culture supernatant. It was performed under reducing conditions as described before [27,28] using a 12.5% polyacrylamide (Rotiphorese® Gel 30 [37.5:1], Carl Roth, Karlsruhe, Germany) separation gel. Five µg purified antibodies were mixed with 4x concentrated sample buffer Roti®-Load 1 (Carl Roth, Karlsruhe, Germany), denatured for 10 min at 99°C, and loaded onto the gel. Electrophoresis was carried out at 120 V for 90 min. Proteins were stained with Coomassie Brilliant Blue (Carl Roth, Karlsruhe, Germany) and documented with the ChemiDoc^TM^ MP Imaging System (Bio-Rad, Munich, Germany).

### K_d_ determination using MicroScale Thermophoresis (MST)

Generated IgG-antibodies were examined for their binding affinity. Therefore, the dissociation constant (K_d_) was measured using MicroScale Thermophoresis (MST) technology [29]. Briefly, 10 µM antigen was coupled using the His-Tag Labeling Kit – RED-tris-NTA 2nd generation (Nanotemper, Munich, MO-L018) according to the manufacturer’s instructions to a red dye that changes fluorescence intensity upon interaction with the antibody due to changes in the chemical environment. This change is directly related to the amount of bound ligand and is enhanced by a precise, laser-induced temperature increase. Therefore, 40 nM of antigen-fluorophore mix was added to 13 µM antibody and diluted serially 1:2. The mixture was loaded on capillaries, and the fluorescence change was measured using the NanoTemper Monolith (NanoTemper, Munich, Germany).

### Statistics

Statistical analysis of the experimental data was performed by using the student´s t-test calculator software from GraphPad Inc. (GraphPad Prism 5, San Diego, CA, USA). P-values of less than 0.05 were considered significant. Data from flow cytometry were analyzed using the FlowJo^TM^ Software v10 (BD, Ashland, USA).

### Ethics statement

This study was carried out in strict accordance with the recommendations in the Guide for the Care and Use of Laboratory Animals of the National Institutes of Health. Our study was approved by the Ethics Committee of the Brandenburg State Office for Occupational Safety, Consumer Protection and Health of the State of Brandenburg under the number 2347-16-2023-32-E. All work performed on the animals was carried out by qualified personnel and complied with the legal guidelines of the Brandenburg State Office for Occupational Safety, Consumer Protection, and Health.

## Results

### De-iniDCs display a more homogeneous cell population than BM-DCs

**Fig 3:**
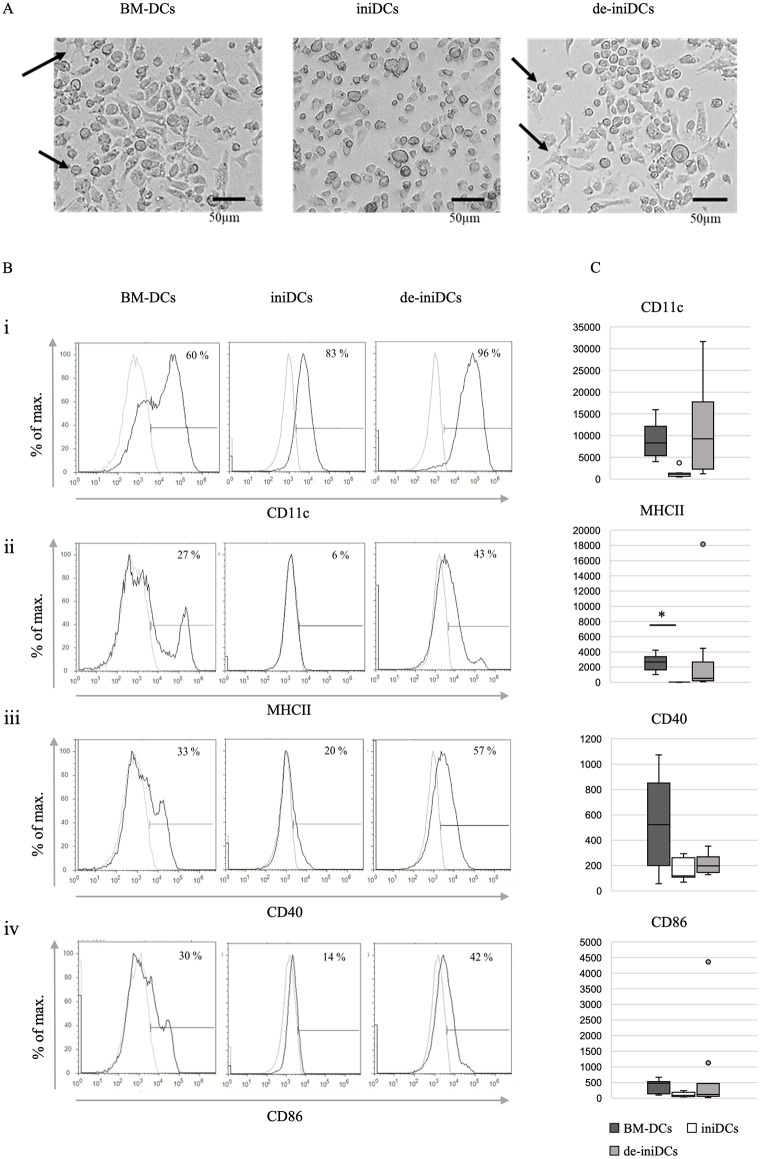
Morphology and flow cytometry analysis of immature BM-DCs, iniDCs and de-iniDCs. **(A)** Microscopic images from immature BM-DCs isolated from a CB57Bl/6 mouse (left panel), iniDCs after 7 days stimulation with Dex/Dox (middle panel), and de-iniDCs after 3 days of deinduction (right panel). Upon deinduction, de-iniDCs display an adherent phenotype as well as typical dendritic extensions indicated by arrows. During their immortal period, iniDCs lose typical dendritic morphology, become round-shaped, and pass over in suspension. (10 x magnification, bar = 50 µm) **(B)** Respectively, 5 x 10^5^ BM-DCs, iniDCs, and de-iniDCs were stained with fluorochrome-conjugated antibodies against specific dendritic surface markers CD11c, MHC II, CD40, and CD86 and analyzed by flow cytometry. Dead cells were removed by PO-PRO^TM^-1 Iodide staining. Experiments were performed 3 times independently. Given is the result of one representative experiment. **(C)** Displayed are the geometric means (MFI) of the cell surface markers, with the background signal subtracted. dark grey = BM-DCs, white = iniDCs, light grey = de-iniDCs, (n = 6), two-tailed t-test (p < 0,05 = *; p < 0,01 = **; p < 0,001 = ***).

Hematopoietic stem cells from double transgenic irtTA-GBD/T-Ag mice were isolated and differentiated into dendritic cells *in vitro* (BM-DCs, Fig 3A, left panel). These cells were then stimulated with dexamethasone (Dex) and doxycycline (Dox) to induce an immortal state. After another 7 days of stimulation, induced immortal dendritic cells (iniDCs) lost their specific dendritic morphology as shown in the middle panel of Fig 3A. Cells were smaller, round-shaped, grew in suspension and dendritic extensions were lost. When the Dex/Dox stimulus was removed for 3 days, de-iniDCs restored their dendritic characteristics and displayed the phenotype of conventional BM-DCs (Fig 3A, right panel) with dendritic extensions and became more adhesive again.

Altered expression patterns of DC-specific cell surface markers were confirmed by FACS analysis. As an overall DC-marker, cells were stained with an anti-CD11c antibody conjugated to PE. Initially, only 60% of BM-DCs expressed CD11c, whereas 40% did not express this DC marker (Fig 3B, i, left panel). When cells were transferred into the immortal period by stimulation with Dex/Dox for 7 days, the CD11c-expression increased by about 23% up to 83% for the iniDCs (Fig 3B, i, middle panel). The CD11c^-^ cell population seen in the BM-DC population almost vanished, and the expression intensity of CD11c^+^ cells decreased by one potency to 10^4^. After removing the immortality stimulus, the CD11c expression intensity increased as well as the number of CD11c^+^ cells to 95.6% (Fig 3B, i, right panel).

Analyses of other specific dendritic surface receptors like MHC II and the important costimulatory molecules CD40 and CD86 displayed a similar pattern. Upon induction of immortality, iniDCs first reduced the expression of MHC II from 27% to 6% (Fig 3B, ii, middle panel), of CD40 from 33% to 20% (Fig 3B, iii, middle panel), and of CD86 from 30% to 14% (Fig 3B, iv, middle panel). Three days after deinduction, the de-iniDCs restored an even more homogeneous expression pattern of all receptors compared to the BM-DCs. The MHC II^+^ cell population increased to 43%, the CD40^+^ cell population to 57%, and the CD86^+^ cells increased to 42% (Fig 3B, right panels). This was also confirmed by the mean fluorescence intensities (MFI) of the different surface markers (3C).

### IniDCs are vivid during long-term culture and regain their proliferation capacity

**Fig 4:**
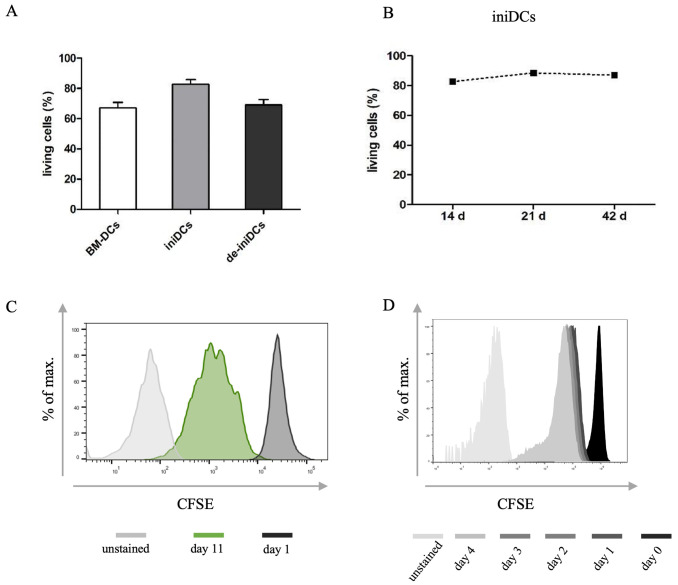
Vitality and proliferation analysis of iniDCs. **(A)** Respectively, 5 x 10^5^ BM-DCs, iniDCs, and de-iniDCs were stained for CD11c and PO-PRO-1 Iodide to define the amount of living CD11c^+^ cells by flow cytometry. After 9 days only 67% (±3.1%) of BM-DCs were alive, 7 days after Dex/Dox stimulation iniDCs displayed 83% (± 2.5%) living cells, and upon deinduction of 3 days vitality decreased to 70% (± 3.8%) of de-iniDCs, n = 3 **(B)** Vitality of iniDCs was stable for 42 days. After 21 days, 88% (± 2.5%, n = 3) and after 42 days, 87% of the CD11c^+^ cells were alive. **(C)** For proliferation analysis best results were achieved by staining 35 x 10^5^ iniDCs with 10 µM CFSE in PBS only. CD11c^+^ cells were analyzed at day 1 (black) and 11 (green) using the BD FACSDAria III^TM^ as well as an unstained control (grey). (D) de-iniDCs were stained with CFSE and measured at day 0, 1, 2, 3 and 4 as well as an unstained control.

Normally, *in vitro* generated BM-DCs lose their proliferation capacity upon development, and fully differentiated BM-DCs only survive a few days in culture until they die or lose their specific DC properties. Live and dead staining with PO-PRO-1 Iodide revealed 67% (± 3.1%) alive CD11c^+^ BM-DCs 9 days after isolation (Fig 4A). Upon induction of immortality, the CD11c^+^ living cell population of iniDCs increased to 83% (± 2.5%, Fig 4A) and was stable at almost 90% over 42 days after isolation (Fig 4B). By removing the immortalization stimulus for 3 days, de-iniDCs lost their proliferation capacity again (Fig 4D) and living CD11c^+^ cells decreased to 70% (± 3.8%, Fig 4A). The longer de-iniDCs were kept in culture the more cells underwent apoptosis.

Since iniDCs were stable in long-term culture and could be expanded easily, their proliferation capacity was tracked using the cell division dye carboxyfluorescein succinimidyl ester (CFSE). For proliferation analyses, iniDCs were stained with either 5 µM, 7.5 µM, or 10 µM CFSE in PBS or PBS/NCS 5%. The best results were achieved by staining cells with 10 µM CFSE in PBS as depicted in Fig 4C. After 11 days, three distinct populations were visible and a fourth was rising. IniDCs displayed a cell division rate of approximately 72 h.

### De-iniDCs mature upon stimulation with antigens and are a more homogeneous cell population than BM-DCs

Upon deinduction for only 3 days, de-iniDCs recovered typical dendrites and surface receptors but lost the ability of cell division. To see if long-term cultured immortal DCs were useful for an *in vitro* immunization, the Dex/Dox stimulus was removed, and it was analyzed if the de-iniDCs were still able to phagocytose, process, and present antigens as BM-DCs would do.

**Fig 5:**
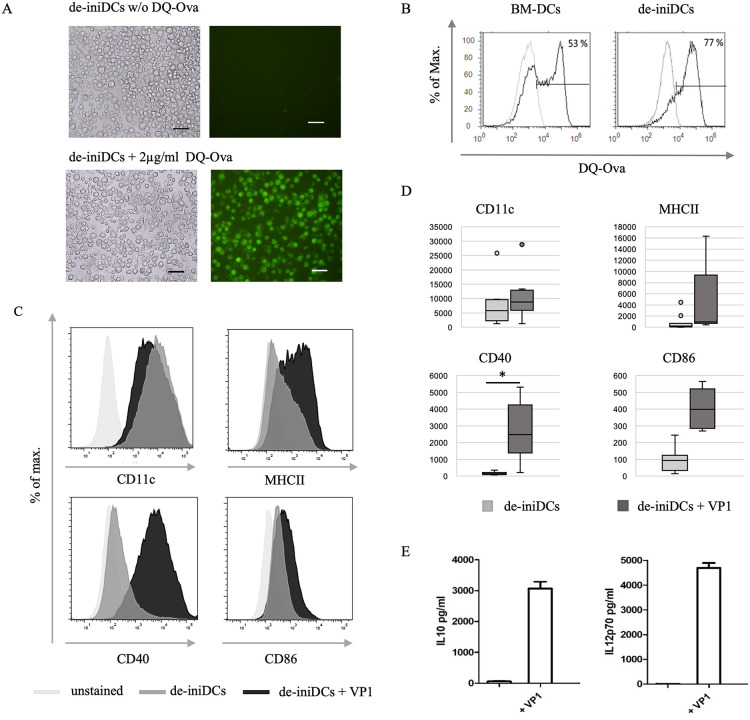
Antigen processing and maturation of immortal dendritic cells. **(A)** 5 x 10^5^ de-iniDCs were stimulated with or without 2 µg/ml DQ-Ova for 60 min at 37°C and measured by fluorescence microscopy using the BZ-800 Biozero (10 x magnification, bar = 50 µm), **(B)** 1 x 10^5^ C57Bl/6 BM-DCs and de-iniDCs were challenged with 1 µg/ml DQ-Ova for 60 min at 37°C (black) and analyzed by flow cytometry using the Attune^®^ Acoustic Focusing Cytometer. Controls are depicted in grey. Given is the result of one representative experiment out of three. **(C)** After maturation of the de-iniDCs with VP1, respectively 5 x 10^5^ cells were stained with fluorochrome-labeled antibodies against maturation markers CD11c, MHC II, CD40, or CD86. To exclude dead cells 1 µg/ml PO-PRO-1^TM^ Iodide was added before analysis using the Attune® Acoustic Focusing Cytometer. Unstained control is in light grey, immature DCs in dark grey, and mature DCs are in black. Given is one representative result out of three. **(D)** Displayed are the geometric means (MFI) of the different surface receptors before and after maturation with VP1. The background signal was subtracted. (n = 8), two-tailed t-test (p < 0,05 = *; p < 0,01 = **; p < 0,001 = ***) **(E)** 1x10^6^ de-iniDCs were matured with 15 µg/ml VP1 for 24 h, and supernatant was analyzed for IL-10 and IL-12p70 by ELISA. (n = 3).

To analyze antigen capture and processing, BM-DCs and de-iniDCs were initially challenged with self-quenched ovalbumin (DQ-Ova). After intracellular proteolytic cleavage, a signal was detected by fluorescence microscopy (Fig 5A) and flow cytometry (Fig 5B). After 60 min of stimulation, 77% of the de-iniDCs were able to engulf and process DQ-Ova (Fig 5B, right panel), whereas only 53% of BM-DCs displayed a DQ-Ova^+^ population (Fig 5B, left panel). Additionally, in flow cytometry, BM-DCs showed a distinct population that was not able to process DQ-Ova at all, which significantly decreased after the immortal period.

After proving that the de-iniDCs are still able to engulf and process antigens, their maturation with our model antigen VP1 was performed for 20−22 h, and cell surface receptors were analyzed by flow cytometry (Fig 5C). For CD11c expression, the de-iniDCs displayed a stable signal before and after VP1 maturation (Fig 5C). Upon maturation with VP1, the fluorescence intensity of MHC II and CD40 increased twofold and for CD86 by one-fold. The signal for CD40 expression showed the most striking difference between the immature and mature state of our *in vitro* generated immortal DC populations (Fig 5C, lower left panel). This was also confirmed by the MFI, which increased upon maturation with VP1 (5D). Additionally, when matured with VP1, de-iniDCs released significantly elevated levels of important cytokines like interleukin (IL) 12 and IL-10. Upon antigen stimulation, IL-12p70 and IL-10 secretion increase up to almost 5000 pg/ml and 3000 pg/ml, respectively (Fig 5E).

Given the results, our *in vitro* generated de-iniDCs showed a comparable high potency to engulf and process antigens as well as to mature after antigen uptake, as conventional BM-DCs generally do. Further, we could show that the de-iniDC population is more homogeneous than a freshly isolated bone marrow-derived DC culture. When stimulated with Dex/Dox, iniDCs regained the ability to proliferate and could be kept in culture for up to several months but lost their typical dendritic morphology and reduced the expression of immunologic surface receptors.

### Matured de-iniDCs start an *in vitro* immune cascade with antigen-specific antibody production

**Fig 6:**
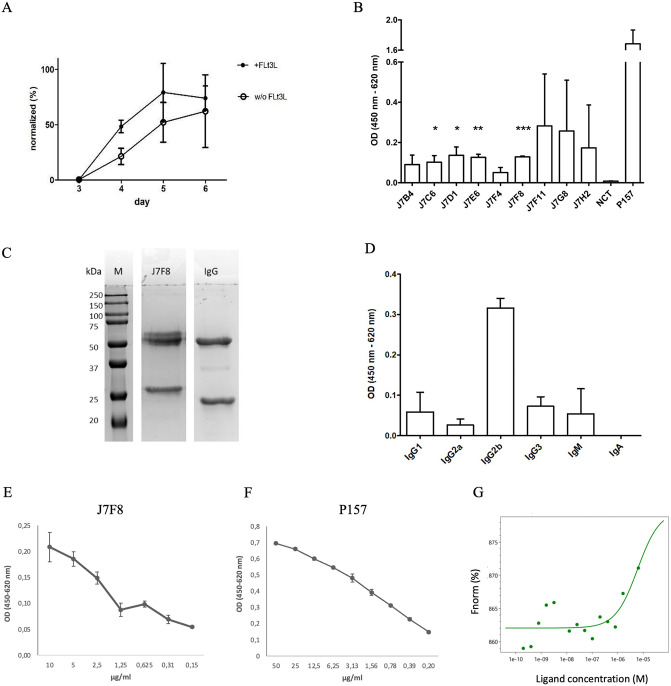
Antigen-specific antibody production of hybridoma cells and isotype of purified antibody. De-iniDCs, T, and B Lymphocytes were cultivated in an IVI with or without Flt3L. After 7 days, immune cells were fused to myeloma cells and selected by HAT-selection. Surviving monoclones were expanded and their supernatants were analyzed for antigen-specific antibody production. **(A)** Unspecific IgG antibody production of IVI supernatant. Displayed are the mean and SD of the normalized OD after 3 (n = 4),4 (n = 4),5 (n = 3), and 6 (n = 3) days of coculture measured by ELISA. **(B)** 9 monoclones were selected and expanded to a 12-well cell culture dish. The supernatant was analyzed for VP1-specific antibody production (n = 3, NCT = negative control, P157 = anti-VP1-IgG antibody generated by standard hybridoma technology). Statistical significance is indicated, *(P < 0.05), **(P < 0.01) and ***(P < 0.001). **(C)** After affinity chromatography purity of monoclonal antibody J7F8 was determined by SDS-PAGE (12,5% PAA, M = Precision Plus All Blue (Bio-Rad, California, USA). **(D)** Additionally, the isotype of J7F8 was determined by ELISA (n = 3) **(E)** as well as a dilution series of the antibody (n = 3). **(F)** Furthermore, an ELISA test was used to determine a dilution series of the P157 antibody (n = 3). **(G)** A K_D_ of 4.52 µM (SD ± 1.5) of the *in vitro* generated J7F8 was measured using MST-technology (n = 2).

To prove if the de-iniDCs are suitable for our *in vitro* immunization protocol, we challenged them with VP1 and cultivated them together with naïve T and B lymphocyte populations in different ratios for 7 days with or without Flt3L. Supernatants were checked for IgG antibody production after 3, 4, 5, and 6 days. After 4 days of co-culture, IgG antibodies were detectable (Fig 6A). When cells were cultured only in RPMI complete medium supplemented with rmGM-CSF, antibody production increased steadily and almost doubled every day. Lymphocytes that were cultured in RPMI complete medium supplemented with rmGM-CSF and Flt3L produced a higher concentration of antibodies, but from day 5 to day 6, IgG production did not increase anymore. On day 7, immune cells were fused with myeloma cells, and after HAT selection, single hybridoma clones were screened for antigen-specific antibody production. After HAT selection of VP1 immunized cells, 20 clones showed an antigen-specific signal when tested by ELISA. From those, 9 were monoclonal (Fig 6B) and 3 stable and well-growing monoclones (J7C6, J7F8, and J7H2) were expanded. The supernatant was checked constantly for antigen-specific antibody production. The supernatant of J7F8 was collected and purified by protein A affinity chromatography. Purity and IgG2b isotype were determined by SDS-PAGE and ELISA (Fig 6C and 6D). Additionally, a K_d_ of 4.52 µM (SD ± 1.5, Fig 6G) was determined using MST technology. In a dilution series the *in vitro* generated J7F8 displayed best binding property at concentration from 10 to 5 µg/ml with an OD of 0.2 (Fig 6E). In contrast, the *in vivo* generated anti-VP1 IgG antibody P157 already showed ODs of 0.2 in an ELISA at concentrations of 0.39 µg/ml (Fig 6F).

**Fig 7:**
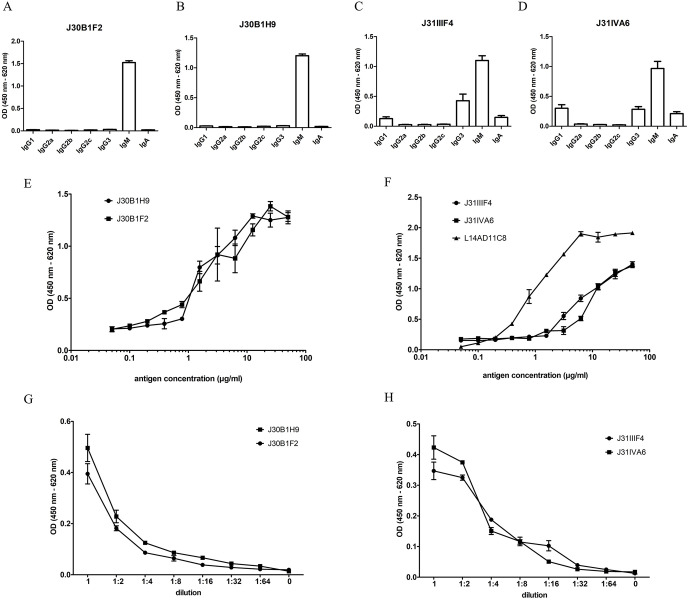
Antibodies generated with *in vitro* immunization (IVI). De-iniDCs were either stimulated with pAS6 or N-Protein and used as antigens in the IVI protocol. After HAT selection of the IVI-pAS6, the two monoclonal hybridomas J30B1F2 **(A)** and J30B1H9 **(B)** were detected to produce antigen-specific IgM antibodies, as well as the two monoclones J31IIIF4 **(C)** and J31IVA6 **(D)** from the IVI-N protein. **(E)**, **(F)** IgM antibodies in the supernatants were further analyzed in an ELISA with an antigen dilution series from 50 µg/ml to 0,05 µg/ml. L14AD11C8 = an anti-N-Protein-IgG antibody generated by standard hybridoma technology **(G)**, **(H)** A serial dilution of the antibody-containing supernatants was performed in an ELISA using 5 µg/ml coated antigen.

To prove the IVI protocol with other antigens, we used the antigen pAS6, which is a peptide sequence expressed with a glutathione-S-transferase (GST)-tag, and the recombinant nucleocapsid protein of SARS-CoV-2 (N protein). For hybridomas derived from the IVI with pAS6, two antigen-specific monoclones J30B1F2 and J30B1H9 could be established, and antigen-specificity was detected by ELISA screening. Both hybridomas secreted antibodies with the IgM isotype (Fig 7A and 7B). When analyzed by an ELISA with a serial dilution of the antigen, 50% of the IgM antibodies secreted by J30B1F2 were bound at a pAS6 concentration of 87.04 µM (SD ± 24.71) (Fig 7E). For J30B1H9 the dilution showed a 50% binding at a pAS6 concentration of 123.08 µM (SD ± 37.45). In Fig 7G, both IgM antibodies were serially diluted on a fixed antigen concentration. Both antibodies showed similar binding behavior and optimal antigen-specific signals for undiluted or 1:2 diluted culture supernatant.

For the N protein stimulated IVI approach, the cells were fused to transgenic SP2/0-Ag14 myeloma cells to sort antigen-specific antibodies using *selma* technology [24]. From 98 sorted single cells, 39 monoclones survived and were tested for their N protein-specific antibody production in ELISA. The two hybridoma clones J31IIIF4 and J31VIA6 stably produced N protein-specific IgM antibodies (Fig 7C and 7D) and were selected for further analyses. Fig 7F showed that 50% of the IgM antibodies secreted by clone J31IIIF4 were bound at an N protein concentration of 145.2 µM (SD ± 26.65). For clone J31VIA6, the 50% signal could be detected at an N protein concentration of 160,13 µM (SD ± 35.62) (Fig 7F). In addition, both culture supernatants were serially diluted on a constant antigen concentration (Fig 7H). Both clones showed a similar recognition of the antigen with optimal signals at a dilution of 1:2–1:4. Furthermore, the anti-N-protein-IgG antibody L14AD11C8 was included as a positive control (Fig 7F). L14AD11C8 was produced using standard hybridoma technology and showed half-maximal binding of the antigen at 20.28 µM (SD ± 8,86). In a dilution series the antibody already showed highest binding properties at a concentration of 0,24 µg/ml (S2 Fig).

## Discussion

Dendritic cells are the key players in bridging innate and adaptive immunity and initiators of the adaptive immune reaction. As antigen-processing cells (APC), they circulate through the bloodstream to capture foreign antigens, process them, and present antigen fragments via MHC II receptors on their cell surface. They then migrate to the lymph nodes to interact with naïve lymphocytes to activate an immune cascade, which leads to the production of highly specific antibodies produced by B lymphocytes [3]. This process can be mimicked *in vitro*. Former studies of our lab could show the possibility of setting up an *in vitro* immunization method to induce the generation of specific antibodies against different antigens [1]. To transfer this *in vitro* immune reaction to standardized reproducible experimental conditions, continuously growing immune cell populations would be desirable. They could be used as a permanent source, replacing animals as immune cell donors.

To establish stable long-term cultures of DCs, immortal DCs generated from a double transgenic irtTA-GBD/T-Ag mouse [17] were used as a scaffold for coordinated *in vitro* activation of immune cells. Therefore, hematopoietic bone marrow cells from double transgenic mice were isolated and differentiated in the presence of GM-CSF over 7 days to BM-DCs [5]. To induce the expression of the tetracycline-regulated SV40 large T-antigen and overcome proliferation limitations, the cell cultures were stimulated with dexamethasone (Dex) and doxycycline (Dox). During the induced immortal period, these iniDCs showed a constant proliferation rate and lost their typical dendritic extensions. Furthermore, a decrease in the expression of essential surface receptors such as MHC II, CD40, and CD86 was detectable. Being a potent immunomodulatory glucocorticoid, Dex acts as a negative regulator of the immune system, modulates the expression of dendritic receptors, and inhibits the maturation process [30–34]. In addition, Dex inhibits the production of proinflammatory cytokines IL-1β, IL-6, IL-12, and TNF-α [19,34]. Although iniDCs kept their proliferation capacity, they lost their typical dendritic cell properties and consequently are not able to start an immune cascade *in vitro* in this state. In the absence of Dex/Dox, de-iniDCs regained a primary dendritic cell phenotype, stopped expression of the large T-antigen, and therefore lost their immortal stage. Interestingly, the CD11c^+^ population then increased to almost 100%, and the expression of the surface receptors MHC II, CD40 and CD86 was even higher than before the immortal period. That makes de-iniDCs a more homogeneous population of DCs compared to the BM-DCs generated with the standard protocol. This reversible transformation of DCs allows long-term storage and antigen-specific activation when needed.

A former study already showed that de-iniDCs were able to capture and process lipopolysaccharide (LPS), which is a commonly used pathogen-associated molecular pattern (PAMP) and undergo a maturation process [19]. To be a scaffold for the *in vitro* immunization process, those immortal DCs should be able to process and present several different antigens. In the present study, we matured the cells with our model antigens VP1 [20,21], pAS6, and the SARS-CoV-2 nucleocapsid protein [35] and used them for *in vitro* immunization to produce specific antibodies. When stimulated with VP1, de-iniDCs displayed a strong maturation process reflected by the increase of maturation markers and corresponding interleukins (Fig 5D). This effect is triggered by the viral origin of the VP1 which leads to strong immune responses in mice. VP1 is therefore used as a potent carrier for immunizations [20,21,36]. Secreted cytokines act as modulators of the immune response and polarize naïve CD4^+^ T cells to T helper (Th) cells [37,38]. In this process, IL-10 develops an immunosuppressive effect [39–41] and IL-12 a proinflammatory effect [42,43]. IL-12 is released in large quantities upon maturation especially from monocyte-derived (moDCs) and conventional DCs (cDCs) [44], particularly when they encounter pathogenic DNA or TLR agonists. This results in the strong expression of maturation markers and an increased capacity to present antigens [45]. Furthermore, it induces the differentiation of naïve CD4^+^ T lymphocytes to become interferon-gamma (IFN-γ)-producing Th1 effectors in cell-mediated immune responses to intracellular pathogens [46,47]. A positive feedback mechanism arises, as IFN-γ in turn increases IL-12 production [48]. IL-10, on the other hand, suppresses the transcription of IL-12 especially in immature DCs. This results in negative feedback on pro-inflammatory reactions [45,48]. Additionally, IL-10 suppresses the production of pro-inflammatory cytokines such as IL-1, IL-2 and IFN-γ and thus promotes a shift in the immune response away from Th1 towards Th2 polarization or immune tolerance [49,50]. IL-10 production by DCs can also promote the differentiation of regulatory T cells (Tregs) from naïve CD4^+^ T cells, which in turn can enhance the Th2 response or dampen excessive immune reactions. The ratio of IL-10 to IL-12 determines whether naive CD4-positive T cells differentiate into Th1 cells or Th2 cells [51]. This dynamically controls the intensity of the immune response and with regard to the IVI, this leads to a more Th2-primed immune response. Since the resulting immortal DCs still possess all necessary features to interact with immune cells we checked them for their ability to start an immune cascade *in vitro*. Wand et al. [1] already showed a step-by-step *in vitro* activation of immune cells with subsequent antigen-specific antibody production when using BM-DCs from Balb/c mice. In this study, we replaced the original BM-DCs with our immortal DCs. When matured with different antigens, immortal DCs were capable of stimulating T and B lymphocytes and triggering an immune cascade resulting in the secretion of antigen-specific IgM and IgG antibodies.

Only upon stimulation with our model antigen VP1, several anti-VP1 IgG antibody-producing hybridomas could be generated after 7 days. From those, J7F8 was identified as an IgG2b isotype antibody. With a K_d_ value of 4.52 μM, the affinity of J7F8 is lower than that of mAbs generated *in vivo,* which are typically in the range of 10^-7^ to 10^-11^ M [52]. *In vivo* generated antibodies have a longer maturation time and utilize the body’s mechanisms for affinity maturation. This allows them to develop a higher affinity towards their antigen than *in vitro* generated antibodies.

In terms of VP1 as a potent viral antigen, it seems that the B cells were capable of performing class switch recombination (CSR) and switching from an IgM to an IgG2b isotype in this stimulation setup. In previous studies, it was shown that mouse splenic B cells can undergo robust CSR in culture on day 3 when treated with LPS or anti-CD40 antibodies [53]. LPS is triggering Toll-like receptor (TLR) 4, which is described as mandatory to induce CSR *in vitro* [53]. VP1 is a major coat protein of the hamster polyomavirus and could therefore be a potent stimulator of TLR4, inducing CSR *in vitro*.

Furthermore, we cultivated the cells together with Flt3L, IL-21, and SEA. Flt3L is a growth factor boosting the growth of splenic DC populations and increasing their ability to engulf and present antigens to T cells [54]. IL-21 is a cytokine known for a strong and sustained activation of STAT3, which is critical for B and T cell differentiation [55]. It also activates STAT1, which leads to an activation of T, B, and conventional DCs. Moreover, STAT1 plays a role in sustaining Ig production and fosters plasma cell generation [56]. SEA is a potent inducer of CD4^+^ Th2 type responses, important to increase B cell activation by releasing IL-4, IL-5, and IL-13 cytokines. When using SEA in combination with a vaccine, Th2-type cytokine responses were increased [57].

In an activation setup stimulated with the N protein and pAS6 only IgM-producing hybridomas could be detected. In the course of a SARS-CoV-2 infection, studies have shown that IgM antibodies were detectable for the first time after approximately 5 days and IgG antibodies after approximately 14 days [58]. The cultivation conditions should therefore be adapted in further studies to see whether a prolonged cultivation results in IgG production. In the case of pAS6, only 3 clones survived the fusion process, from which 2 clones produced antibodies. Hybridoma cells, in general, are very sensitive to their culture environment, which includes the fusion and HAT selection process. Especially in the early onset after fusion and HAT selection, the cells are unstable and often stop growing or producing the desired antibody. However, the IVI approach described in this study is based on the cell number of 7 x 10^5^ naïve B cells. The standard hybridoma technology uses splenocyte fractions from the whole spleen, which contains around 100 million cells of which 18–20% are B cells in C57Bl/6 mice [59].

To further increase the efficiency in selecting antigen-specific antibody-producing hybridomas, we used the novel hybridoma selection strategy s*elma* [24]. The efficiency of conventional hybridoma technology is relatively low, as only a small fraction of the input B cells ultimately give rise to antigen-specific, antibody-producing hybridomas following cell fusion. After HAT selection, hybridoma populations typically require labor-intensive clonal isolation via limiting dilution, which is both time-consuming and yields a high proportion of polyclonal wells. These must subsequently be re-screened and subcloned to obtain genetically stable monoclonal lines. The overall process is not only inefficient but also imposes significant stress on the often-fragile hybridoma cells, resulting in prolonged timelines of several weeks to months before stable, antigen-specific monoclonal antibody-producing cell lines are established. During *selma*, antigen-specific antibody-producing cells are directly labeled and can be immediately cultivated in single-cell culture. This reduces the screening time to one day and greatly increases the output of positive stable hybridomas. In addition, hybridomas can be selected directly based on their secreted isotope. The enrichment of antigen-specific hybridomas by FACS was performed with an IgM- and IgG-specific antibody capture matrix. Since the number of antigen-specific hybridomas from one IVI setup was so high, we stopped sorting after 98 monoclonals and kept the remaining cells for further analysis. From those 98 antigen-specific monoclonals, 39 could be established in cell culture. In total, 10 of them started growing and produced specific IgM antibodies. The combination of an *in vitro* immunization with a mammalian cell surface display system like *selma* has several benefits. First, it allows a fast selection of antigen-specific hybridomas and increases the output significantly when compared to standard limited dilution technology. Secondly, the IVI approach described in this study enables the use of *in vivo* pathological or toxic antigens and needs lower concentrations of antigens than *in vivo* immunizations.

Our immortal DC approach can function as the first step in an *in vitro* immunization process. They can be kept in long-term culture, frozen and thawed, de-induced, and then used in the *in vitro* immunization process to produce antigen-specific monoclonal antibodies of different isotypes. Furthermore, our immortal DCs are a more homogeneous conventional DC population than the currently developed BM-DCs from standard protocols. This can be a great benefit for further immunogenicity studies, vaccine testing, or T cell reactivity studies. Therefore, the use of laboratory animals could be drastically reduced in favor of a more defined *in vitro* environment.

To establish a complete and universal artificial immune reaction for murine cells also T and B lymphocytes need to be immortalized. Ongoing experiments are in progress to see if those cell populations from double transgenic irtTA-GBD/T-Ag mice can also be reversibly transferred into an immortal period. Additionally, T and B cells are currently modified with an oncogene by lentiviral transduction to reach an immortal state and be kept in long-term culture.

Current results from our lab using primary CB57Bl/6 B cells showed an exclusive B cell activation that might be sufficient to produce antigen-specific monoclonal antibodies *in vitro* [60]. This workflow will be adapted to the B lymphocytes isolated from the double transgenic irtTA-GBD/T-Ag mice to establish a fast and complete *in vitro* approach for the generation of antigen-specific monoclonal antibodies.

Our study aimed to create a basic protocol for an alternative method of murine antibody production in the sense of the 3Rs (Replace, Reduce, Refine). This must be further optimized and adapted regarding the lower affinity and IgM isotypes of the currently produced antibodies. While the *in vitro* immunization (IVI) approach presented in this study successfully replicates key aspects of murine antibody production, it does not yet fully replace *in vivo* immunization. Major limitations include the absence of complex structures such as secondary lymphoid organ architecture, the germinal center microenvironment, and influences from the microbiome, all of which are essential for affinity maturation and isotype switching. While antigen-specific monoclonal antibodies can be produced *in vitro*, they often show lower affinity and limited class switching. *In vivo*, functional maturation and selection primarily occur in germinal centers, resulting in high-affinity, long-lived antibody responses [61]. This selection process is highly dependent on time- and cytokine-coordinated signaling within a complex, three-dimensional microenvironment involving follicular dendritic cells and T follicular helper cells. The immune cell repertoire *in vivo* is also significantly larger and more diverse than within an IVI. In fact, traditional *in vivo* immunization protocols frequently yield high-titer, high-affinity monoclonal and polyclonal antibodies, owing to the intrinsic efficiency of these physiological selection mechanisms. Nevertheless, IVI systems offer several key advantages. Under optimized conditions, including appropriate antigen presentation, cytokine supplementation, and use of naïve antigen-inexperienced lymphocytes, they can generate antigen-specific antibodies with comparable specificity to those obtained *in vivo*. Recent studies indicate that IVI antibodies can reach similar affinities under optimized conditions [62]. A major benefit of IVI systems lies in their temporal efficiency. *In vitro* immunization can lead to detectable antibody secretion within days, vastly accelerating discovery timelines. In contrast, *in vivo* immunizations typically require several weeks to months. Finally, while IVI systems may inherently produce a narrower range of epitope binders, growing evidence suggests that they are capable of generating functionally diverse antibody pools. Future improvements in mimicking the germinal center environment *in vitro* may enhance the quality and diversity of IVI-generated antibodies. Further studies of our research group dealt with the replication of an artificial lymph node and could already show that our IVI approach also works in the three-dimensional system [63]. As described in Engel et al. [63], the number of cells in the IVI could be increased by using this 3D structure. A fusion of these methods could lead to an improvement in murine antibody production with acceptable affinity.

## Supporting information

S1 Fig Raw SDS-PAGE image of J7F8. After affinity chromatography purity of monoclonal antibody J7F8 was determined by SDS-PAGE (12,5% PAA, M = Precision Plus All Blue (Bio-Rad, California, USA). Dotted lines indicate where the image was cut. Areas marked with an X have been removed.
(TIF)

S2 Fig Dilution series of the *in vivo* generated mouse anti-N Protein IgG antibody L14AD11C8. The antibody L14AD11C8 was serially diluted on a fixed concentration of 5 µg/ml antigen in an ELISA. (n = 3).
(TIF)

S3 Fig CFSE staining of iniDCs. For proliferation analysis, best results were achieved by staining 35 x 10^5^ iniDCs with 10 µM CFSE in PBS only. CD11c^+^ cells were analyzed at day 1 (black), 6, 8, 11 and 15 (green) using the BD FACSDAria III^TM^ as well as an unstained control (grey). (n = 3).
(TIFF)

S1 Data Minimal data. (XLSX)
