# Supplementary figures and images for: Inducible immortalized Dendritic Cells enable antigen-specific antibody production in a murine in vitro Immunization model
Source: PLoS One. 2026 Jan 2;21(1):e0339883. doi: 10.1371/journal.pone.0339883 (PMC12758701; doi:10.1371/journal.pone.0339883)

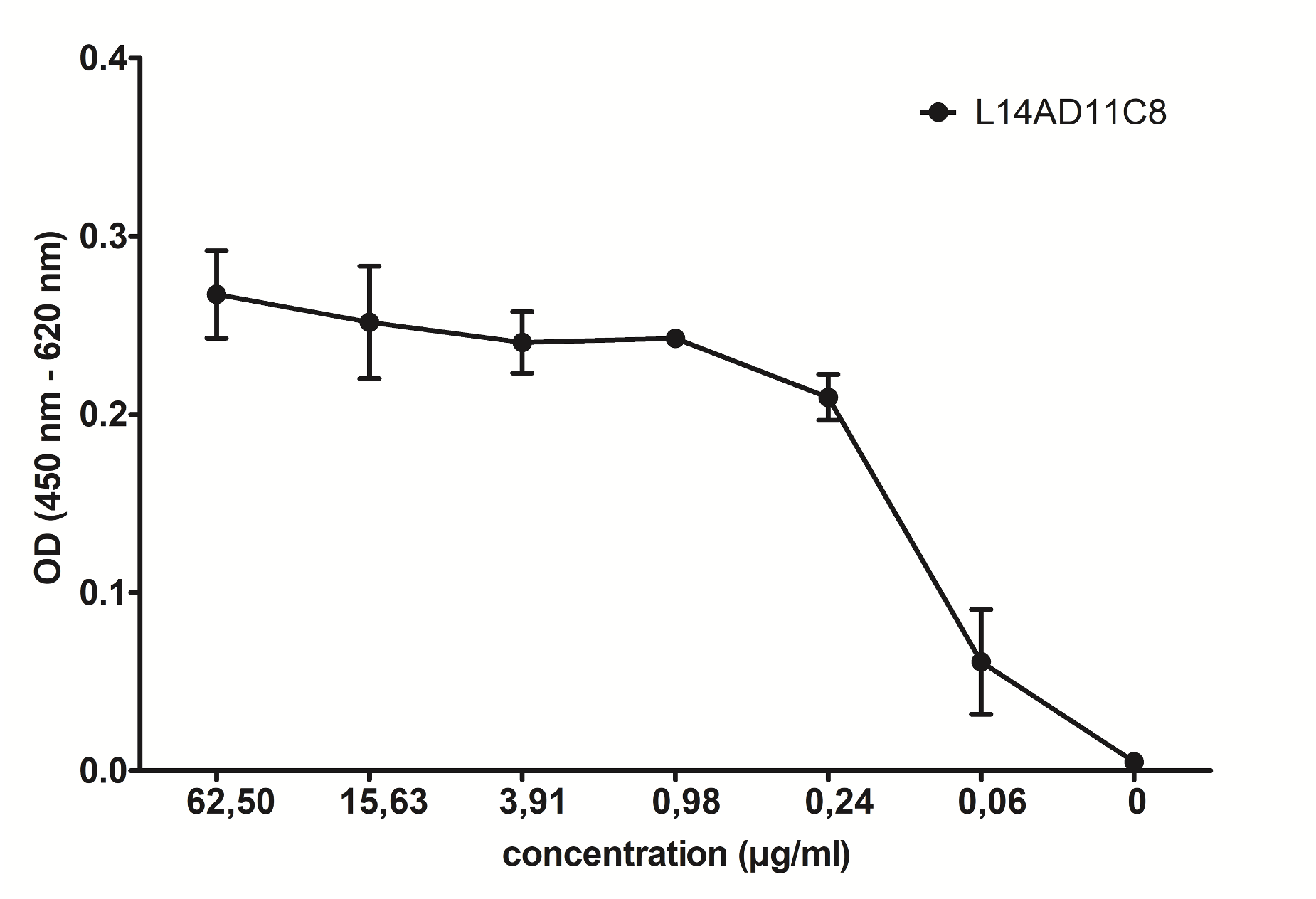

Supplement: S2 Fig — The antibody L14AD11C8 was serially diluted on a fixed concentration of 5 µg/ml antigen in an ELISA. (n = 3). (TIF) [file pone.0339883.s002.tif]

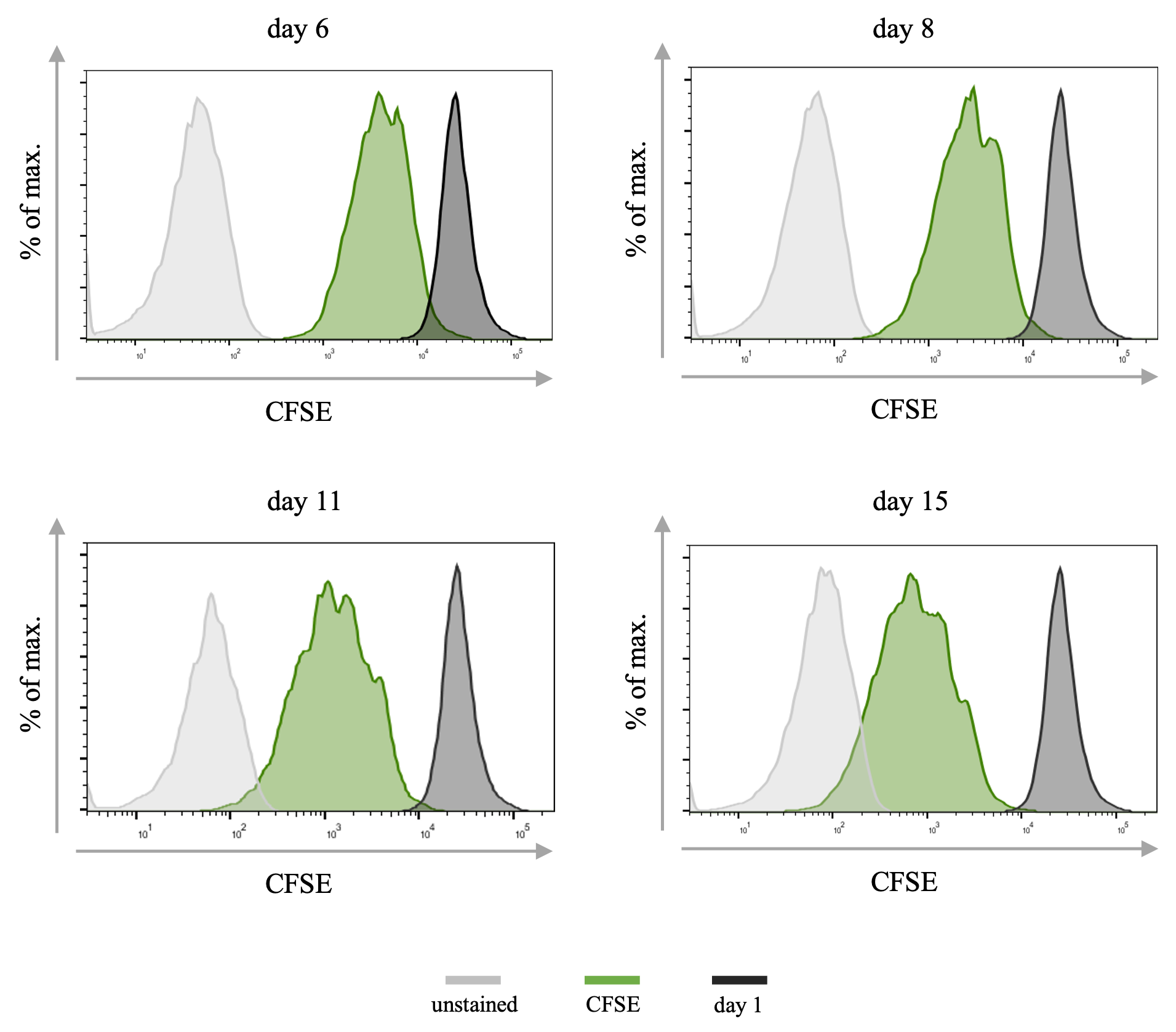

Supplement: S3 Fig — For proliferation analysis, best results were achieved by staining 35 x 105 iniDCs with 10 µM CFSE in PBS only. CD11c+ cells were analyzed at day 1 (black), 6, 8, 11 and 15 (green) using the BD FACSDAria IIITM as well as an unstained control (grey). (n = 3). (TIFF) [file pone.0339883.s003.tiff]
